# Skin Testing to Identify Safe Drugs for Patients with Rocuronium-Induced Anaphylaxis

**DOI:** 10.1155/2020/8163620

**Published:** 2020-01-28

**Authors:** Manzo Suzuki, Hajime Kawase, Azusa Ogita, Hiroyasu Bito

**Affiliations:** ^1^Anesthesiologist, Yatsu Health Hospital, Chiba, Japan; ^2^Department of Anesthesiology, Musashikosugi Hospital Nippon Medical School, Kawasaki, Kanagawa, Japan; ^3^Department of Dermatology, Musashikosugi Hospital Nippon Medical School, Kawasaki, Kanagawa, Japan

## Abstract

Among patients who develop anaphylaxis during anesthesia, anaphylaxis caused by a neuromuscular blocking agent has the highest incidence. In patients who developed IgE-mediated anaphylaxis, and cross-reactivity among NMBAs is a concern in subsequent anesthetic procedures. We present a patient who developed rocuronium-induced anaphylaxis in whom the skin prick test (SPT) and intradermal test (IDT) could identify a safe drug to use in the subsequent anesthetic procedure. A 32-year-old female developed anaphylactic shock at the induction of general anesthesia. She recovered by administration of hydrocortisone and epinephrine. Skin tests including the SPT followed by the IDT revealed rocuronium as the drug that caused anaphylaxis and vecuronium as a safe drug to use for the subsequent general anesthesia. She safely underwent surgery with general anesthesia using vecuronium one month after the skin testing. There are not many reports on the effectiveness of the SPT followed by IDT in identifying the causative drug as well as a safe drug to use in the subsequent anesthetic procedure following anaphylaxis during anesthesia. The usefulness of the SPT should be re-evaluated.

## 1. Introduction

Anaphylaxis during anesthesia is uncommon and is sometimes life-threatening. Neuromuscular blocking agents (NMBAs), latex, and antibiotics are among the causes of anaphylactic reaction during anesthesia [1]. In approximately sixty percent of anaphylaxis cases during anesthesia, anaphylaxis is mediated by an NMBA [2]. Although succinylcholine is associated with a high incidence of anaphylaxis [3, 4], the number of reports on anaphylactic and anaphylactoid reaction due to rocuronium has recently been increasing [5].

Administration of an NMBA such as rocuronium can induce an immunoglobulin E- (IgE-) mediated or non-IgE-mediated anaphylactic reaction. In patients who developed IgE-mediated anaphylaxis, cross-reactivity among NMBAs is a concern in subsequent anesthetic procedures. The causative drug is identified by testing such as with the skin prick test (SPT) and intradermal test (IDT). *In vitro* testing, i.e., identification of the specific IgE against NMBA and basophil activation test (BAT), has become available in recently developed procedures [6, 7]. In addition to identification of the drug that caused the anaphylaxis reaction, it is important to determine which drug is safe for subsequent anesthetic procedures. We present a patient who developed rocuronium-induced anaphylaxis in whom the anaphylaxis skin test could identify a safe drug to use in the subsequent anesthetic procedure.

## 2. Case Presentation

A 32-year-old female was scheduled to undergo laparoscopic ovarian cystectomy. Her past history was unremarkable except for the presence of contact allergy to metal, and she had never undergone an anesthesia procedure previously. Her height was 164 cm, and weight was 79 kg. An epidural catheter was placed before anesthesia induction. Povidone iodine was used for skin disinfection, and mepivacaine 0.5% was used for skin infiltration of local anesthetics. During and after placement of the epidural catheter, the patient's condition was stable. General anesthesia was induced by propofol 150 mg, and continuous infusion of remifentanil 0.3 *μ*g/kg/min and rocuronium 50 mg was administered to facilitate tracheal intubation. Anesthesia was maintained by continuous infusion of remifentanil and desflurane. After administration of rocuronium, her entire body began to flush. Even though tracheal intubation was uneventful, the anesthesiologist felt very high resistance during manual bag ventilation. Peak airway pressure increased to 37 cm·H_2_O with a tidal volume of approximately 170 ml. The value of end tidal carbon dioxide tension was approximately 17 mmHg. Her systolic blood pressure decreased to 40 mmHg, and heart rate increased to 170 bpm. Because anaphylactic reaction was suspected, hydrocortisone 300 mg was administered intravenously, and epinephrine 0.2 mg was administered intramuscularly. Starting at about five minutes after administration of epinephrine, the peak airway pressure gradually decreased to 20 cm·H_2_O, and skin flushing seemed to decrease. Her blood pressure and heart rate stabilized to 100 mmHg of systolic blood pressure and 90 bpm of heart rate. Surgery was cancelled. Thirty minutes after recovery from shock state, the train-of-four ratio by neuromuscular monitoring returned to over 90%, and the patient regained consciousness. Her trachea was extubated. We did not measure the serum tryptase level because its measurement is not available at our hospital.

### 2.1. Skin Testing

A dermatologist was consulted and skin testing including the SPT and IDT for rocuronium, vecuronium, propofol, mepivacaine, and midazolam was scheduled. The skin prick test using administration of histamine as a positive control and saline as a negative control was undertaken in the operating room by the dermatologist. The concentrations of the test drugs used in the SPT and IDT are shown in Table 1. All drugs diluted to 1 : 100 in the SPT resulted in no reaction. However, among undiluted agents in the SPT, only rocuronium presented enlargement of flare and wheal, which indicated a positive allergic reaction. Other drugs did not present a positive reaction. The next day, the intradermal skin test was performed to identify whether cross-reaction between rocuronium and vecuronium could be observed. Only vecuronium was examined. Vecuronium, diluted at 1 : 1000, 1 : 100, and 1 : 10, was tested at 20 minute intervals, and no positive reaction was found at any dilution.

Laparoscopic ovarian cystectomy was rescheduled after one month. General anesthesia using vecuronium was safely performed. The surgery was uneventful, and the patient is doing well.

## 3. Discussion

Our patient developed circulatory shock, tachycardia, and bronchospasm with skin flush after induction of general anesthesia, which were suspected to be due to anaphylaxis. In the present case, the severity of anaphylaxis was grade III according to the scale developed by the Societe Francaise d' Anesthesie et de Reanimation (SFAR) [2]. The patient's symptoms were severe, but she recovered by administration of intramuscular epinephrine.

When our patient developed these symptoms, we cancelled the surgical procedure. However, a study on a large series of patients who developed anaphylaxis during anesthesia suggested that there is no difference in anaphylaxis-related outcome between patients in whom the surgical procedure was continued, and patients in whom the procedure was cancelled among cases where immediate recovery from anaphylaxis was obtained [8]. If a patient recovers from shock state, it may be safe to proceed with the procedure.

In the present case, mepivacaine, propofol, remifentanil, rocuronium, and povidone iodine used for disinfection were thought to be among the causative agents because the patient's condition was stable until the induction of general anesthesia. The causative drug was thought to be the drug used for induction of general anesthesia. In the present case, we performed SPT and IDT to identify the causative drug and also a safe drug to use for subsequent anesthesia. In the present case, undiluted rocuronium induced a flare and wheal which is a positive sign in the SPT. In the study of Dhonneur et al. [9], anesthesia-naïve volunteers received SPTs with various concentrations of rocuronium and vecuronium, and the authors found that 50% of the subjects had a positive skin reaction to undiluted rocuronium. And, in intradermal skin testing, non-mast-cell-mediated positive reaction are frequent with rocuronium and cisatracurium [10]. This irritating concentration of drug may induce a false-positive reaction. However, in patients with a history of hypotension and bronchospasm during anesthesia, the positive predictive value of the SPT was 98% [6]. In the same series by Berg et al. investigating cisatracum and rocuronium, no false-positive reaction was observed by undiluted agents in SPT [10]. We performed IDT using 1 : 100 and 1 : 10 dilution of vecuronium according to the guideline by SFAR. The dilution of 1 : 10 in vecuronium was the upper limit of testing concentration. Meters et al. demonstrated that, in healthy volunteers, reactive(false-positive) dilution in vecuronium was 1 : 3.1 and nonreactive(false-negative) dilution was 1 : 31. In the concentration, we have used in our patient, there is still small possibility of false-negative results in IDT. The efficacy of *in vitro* tests such as identification of the specific IgE against NMBA and BAT was proposed in recent studies; however, the negative predictive value is higher in skin testing compared with *in vitro* testing [11–13]. Leysen et al. suggested that quantification of specific IgE antibodies may be valuable in cases where negative skin testing is obtained in patients with a history of NMBA-induced anaphylaxis [6, 7].

In a recent animal study, it was shown that NMBA activates mast cell degranulation through Mas-related G-protein-coupled receptor member X2 (MRGPRX2) activation independent of the presence of IgE antibodies [14, 15]. Activation of this receptor induces a non-IgE-mediated response in NMBA-naïve patients such as a pseudo-allergic reaction. One important note is that in patients possessing this receptor, skin testing can give a false-positive result even though patients do not have IgE antibody. This phenomenon induces relatively mild and transient symptoms in a dose-dependent manner and resembles an anaphylactoid reaction. In the present case, the patient had skin contact allergy to metal. The high probability of the presence of MRGPRX2 in keratinocytes in patients with urticaria indicates that there remains a possibility of pseudoallergic reaction in the present case.

The most remarkable point in this case is that, when a positive result in rocuronium and negative result in vecuronium were found in the SPT, it was worthwhile to proceed with the IDT of vecuronium to confirm the absence of cross-reaction. In the present case, subsequent general anesthesia using vecuronium was uneventful. Fisher et al. [16] reported three patients who developed subsequent allergic reaction to a NMBA that had negative responses on skin testing and IgE testing. They reported cross-reaction between decamethonium and succinylcholine, between pancuronium and alcuronium, and between rocuronium and vecuronium. Chiriac et al. [3] demonstrated that among 92 patients who presented hyperreaction to NMBA, 25 patients received an NMBA in subsequent general anesthesia, and 2 patients developed anaphylaxis with re-exposure to a negative skin-tested NMBA.

The usefulness of the skin prick test in identifying the drug that causes anaphylaxis should be re-evaluated. In patients who develop anaphylaxis during anesthesia, it is important to perform the SPT followed by the IDT not only to identify the causative drug but also to confirm which drug is safe to use in the subsequent anesthetic procedure.

## Figures and Tables

**Table 1:** Concentrations of agents in the skin prick test and intradermal test.

Substance	Skin prick test (mg/ml)		Intradermal test (mg/ml)		
Dilution	1 : 100	Undiluted	1 : 1000	1 : 100	1 : 10
Propofol	0.1	10	0.01	0.1	1
Midazolam	0.05	5	0.005	0.05	0.5
Mepivacaine	0.1	10	0.01	0.1	1
Rocuronium	0.1	10	0.01	0.1	1
Vecuronium	0.1	1	0.01	0.1	1
